# FriiTriga, a triggered controller with an optical beam shutter using a hard disk drive voice-coil actuator

**DOI:** 10.1016/j.ohx.2024.e00542

**Published:** 2024-06-07

**Authors:** Dam-Bé L. Douti, Chérif Ouro-Wassara, Essohana Mabafei

**Affiliations:** Laboratoire Matériaux, Energies Renouvelables et Environnement, Faculté des Sciences et Techniques, Université de Kara, BP 404 Kara, Togo

**Keywords:** Lowcost, Trigger controller, Optical shutter, Optomechanics, Laser

## Abstract

We present in this article the complete setup to build a triggered controller with a mechanical optical shutter. The system is a low cost, Do-It-Yourself, and easy to implement setup with three functionalities: Manual mode, direct mode with TTL signal command and Triggered mode with a TTL signal command. Our setup is primarily intended to be integrated in optical setup where one needs to control the opening time of a light path, but can be used also for any other setup where one wants to send a TTL signal to command another subsystem (in our case the shutter is that subsystem). The shutter used here is hard disk drive voice-coil actuator, which was already demonstrated to have interesting potentialities to be a mechanical shutter.

In the Manual mode, this setup achieves an opening and closing time of 3 ms. In Direct mode with TTL signal command, the setup has a delay response time of 19 ms and a minimum open pulse time of 23 ms. This low cost device which can be made with less than 50€, have similar characteristics with commercial ones which can be twenty times more expensive.

## Graphical abstract

**Figure:**
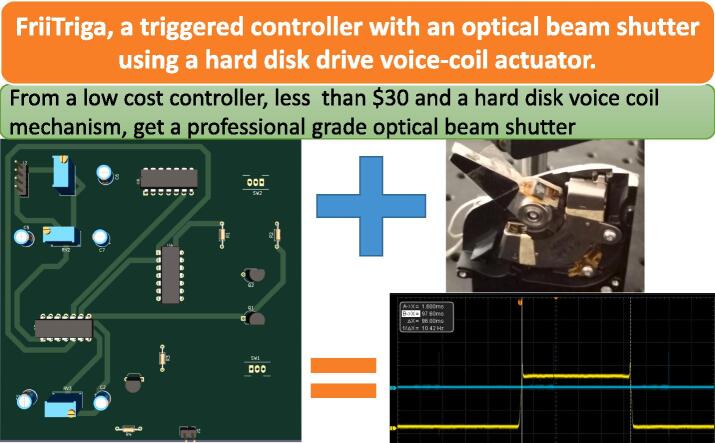

**Specifications table**

**Table:** 

Hardware name	*FriiTriga*
Subject area	• Engineering and materials science • Educational tools and open source alternatives to existing infrastructure • General
Hardware type	• Mechanical engineering and materials science • Light control tool
Closest commercial analog	*There are many commercial optical shutter (Thorlabs, EdmundOptics, Newport, EksmaOptics), but Thorlabs component, Optical Beam Shutter SH05 and its controller KSC101, is the one we compare our device with.*
Open source license	*CERN Open Hardware License (OHL)*
Cost of hardware	*$27.55*
Source file repository	*https://doi.org/10.5281/zenodo.11092585*

## 1 Hardware in context

Laser based experiments requires usually a control of the beam switching time, which can be manual or triggered with another signal. Optical beam shutters are available commercially but costs more than 1000€ (including the controller); as the one from Thorlabs [1], SH05 and its controller KSC101, which is available at 1000€. Some researchers have already proposed to build laser shutters using electromagnetic induction effect [2], [3], hard disk voice coils [4], direct current motors [5] or piezoelectric actuator [6], [7]. Each method has its advantages, but in term of costs, the one based on hard disk voice coil seems to be the cheapest since the core component is easily accessible in any old magnetic hard disk drives. This is the main reason this method has been chosen in this study. Another point also is that the commercial laser shutters offers mainly two functions: manual operation and signal triggered mode (in/out). To our knowledge, there is no article on a “Do It Yourself” purpose which proposed a full controller setup for a triggered laser shutter. The setup proposed in this article is a hardware with three functions, so that it can fully replace a commercial hardware, but for less than 50€.

## 2 Hardware description

• Three modes available: manual, direct, triggered
• Initially designed for light control experiment (such as spectroscopy), so it can be used without any modification for these purpose
• The electronic board controller can be used for any other goals where one needs a triggered TTL signal to control another subsystem

The hardware setup presented here is a fully tunable triggered shutter, which main functionalities are: manual mode, triggered mode with a TTL signal, non-triggered direct mode with TTL. The tuning of the opening time in the triggered mode can be made using a potentiometer on the electronic board. This setup can be useful in optical spectroscopy setup, or any light control setup that needs time triggering.

The system is composed of two parts: the electronic board which is the controller and the mechanical shutter. The controller can be used for any other purpose where one needs time triggering with a 5 V TTL output. The output of the controller is used, in our case, to control a mechanical shutter but it can be used for any other purpose.

### 2.1 Direct mode

**Fig. 1:**
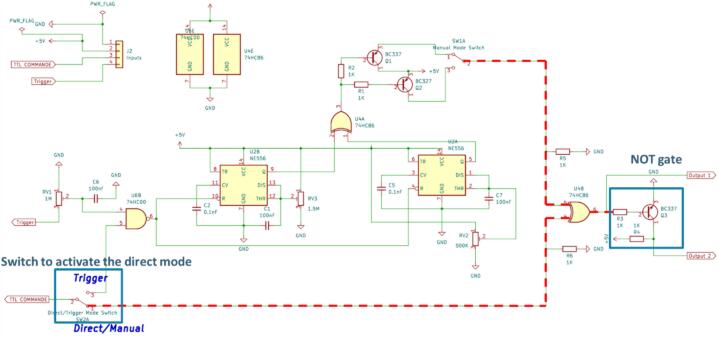
Friitriga controller scheme, showing in red the path of the signal. (For interpretation of the references to colour in this figure legend, the reader is referred to the web version of this article.)

The direct mode can be activated on the electronic board with the switch SW2A as indicated in Fig. 1.

During this mode only the command signal is needed as input for the controller.

When activated, the controller sends directly to the shutter the TTL signal that has been entered as a command. The shutter will then be open during the high state time of the command signal (signal time width).

### 2.2 Triggered mode

This mode is activated by turning the switch SW2A in the opposite direction. During the triggered mode, the controller takes as input the trigger signal and a command signal. The command signal time width ($\tau_{cmd})$ has to be less than or equal to the trigger signal period (T): $\tau_{cmd}\leq T$. The opening time of the shutter here is defined by the potentiometer RV2.

**Fig. 2:**
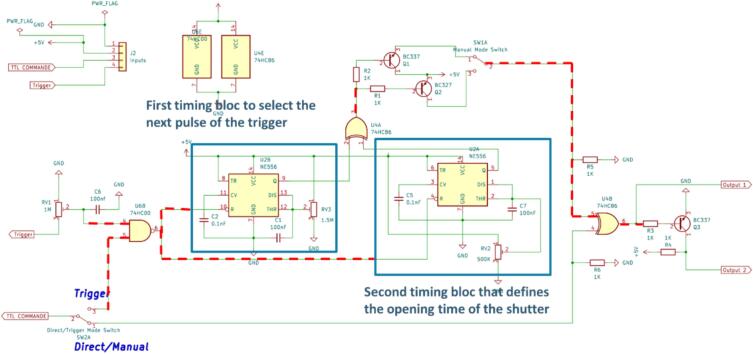
Friitriga controller scheme, showing the signal paths when in triggered mode.

One thing important to notice, is that the triggered mode is intended to align the opening of the shutter with the next pulse of the trigger. That means the trigger signal has to be a periodic signal (Fig. 2).

The tuning of the first timing bloc is made by changing the values of the capacitor C_1_ and the value $R_{1}$ of the potentiometer RV3. This first timing bloc mainly generate a square pulse which time width ($\tau_{1}$) value should be half of the trigger period T. For this purpose, one should choose values so that:

(1) $$1.1\times {\text{R}}_{1}\times {\text{C}}_{1}=\frac{\text{T}}{2}$$

The second timing bloc is intended to define the opening time (OT) of the shutter. This opening time can be expressed in term of number of trigger period: OT=n×T. The tuning of this timing bloc is operated by the capacitor C_7_ and the value $R_{2}$ of the potentiometer RV2. One should choose values so that

(2) $$1.1\times {\text{R}}_{2}\times {\text{C}}_{7}=\left(\text{n}+\frac{1}{2}\right)\text{T}$$

As a case study, let us suppose that the controller is used to trigger a pulsed laser light with repetition rate of 10 Hz. The trigger signal frequency is then equal to 10 Hz (T=100ms). For the choice of the capacitors and potentiometers values, one can chose to setup the controller for an opening time equal one period of the trigger (n=1). This means for the first timing bloc (Equ.1), one should have $1.1\times R_{1}\times C_{1}=50.10^{-3}$ and for the second timing bloc (Equ.2), $1.1\times R_{2}\times C_{7}=150.10^{-3}$. If the values of the capacitors are fixed ($C_{1}=C_{7}=100nF$), one should have then: $R_{1}=\frac{50.10^{-3}}{1.1\times 10^{-7}}=454.5K\Omega$ and $R_{2}=\frac{150.10^{-3}}{1.1\times 10^{-7}}=1.36M\Omega$. For a better tuning of the controller, it’s advised to use potentiometers as done in this study with potentiometers RV2 and RV3: $R_{1}=RV2=500K\Omega$ and $R_{2}=RV3=1.5M\Omega$

### 2.3 Manual mode

**Fig. 3:**
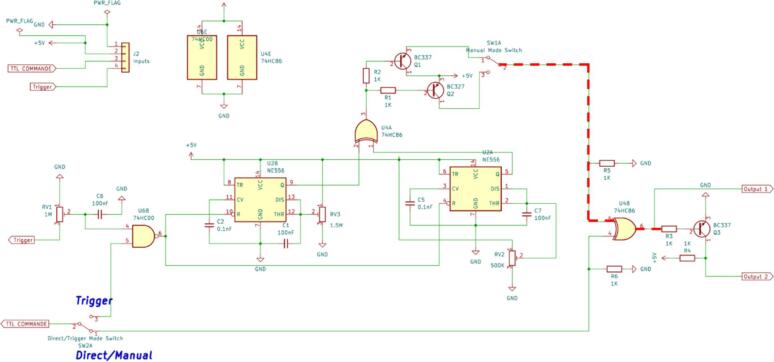
Friitriga controller showing the signal path when in manual mode.

In the manual mode, the controller doesn’t need any input signal. The manual mode is automatically activated when switching the switch SW1A: one position is for closing the shutter and the other one will open it (Fig. 3).

### 2.4 The mechanical shutter

The mechanical shutter is composed of two parts: the driver module and the hard disk drive voice-coil actuator. The driver module converts the TTL signal coming from the controller to an intensity pulse capable of activating the voice-coil actuator. It’s mainly a H-Bridge circuit for motor control. The voice-coil actuator is part of magnetic hard disks; for a better understanding of how a voice-coil actuator works and how to get them from a hard disk, one can read these articles [4], [8], [9] of Landolsi et al., Maguire et al. and Scholten et al.

***Design files***

## 3 Design files summary

**Table:** 

Design file name	File type	Open source license	Location of the file
*FriiTriga_KiCAD_Design.zip*	CAD files of the circuit	*CERN Open Hardware License (OHL)*	https://doi.org/10.5281/zenodo.11092585

The complete files of the Friitriga Controller are provided, and can be edited with the open source software KiCad.

***Bill of materials***

## 4 Bill of materials summary

**Table:** 

Designator	Component	Number	Cost per unit −currency	Total cost − currency	Source of materials
Capacitors	100nF (C_1_, C_6_, C_7_)	3	$1.2	$3.6	https://us.rs-online.com/web
Capacitors	0.1nF (C_2_,C_5_)	2	$1.2	$2.4	https://us.rs-online.com/web
Resistors	1 K	6	$0.15	$0.9	https://us.rs-online.com/web
Integrated circuit	NE556	1	$1	$1	https://us.rs-online.com/web
Integrated circuit	74HC386	1	$0.5	$0.5	https://us.rs-online.com/web
Integrated circuit	74HC00	1	$0.5	$0.5	https://us.rs-online.com/web
Transistor	BC337	1	$0.3	$0.3	https://us.rs-online.com/web
Transistor	BC327	2	$0.3	$0.3	https://us.rs-online.com/web
Miscellaneous	Single row Female 02 Pin Header (CONN-SIL2)	1	$0.10	$0.10	https://us.rs-online.com/web
Miscellaneous	Single row Female 04 Pin Header (CONN-SIL4)	1	$0.10	$0.10	https://us.rs-online.com/web
Miscellaneous	Potentiometer (RV1 – 2 M)	1	$0.15	$0.15	https://us.rs-online.com/web
Miscellaneous	Potentiometer (RV2 – 500 K)	1	$2	$2	https://us.rs-online.com/web
Miscellaneous	Potentiometer (RV3 – 1.5 M)	1	$2	$2	https://us.rs-online.com/web
Miscellaneous	Mechanical switch SW-SPDT	2	$7	$14	https://us.rs-online.com/web
H Bridge driver module	L298N Module	1	$16.9	$16.9	https://www.amazon.co.uk/Bridge-Stepper-Driver-Controller-Arduino/dp/B014KMHSW6/ref = sr_1_4?crid = 2F1SHPZRGUZO7&currency = USD&keywords = l298n + motor + driver&qid = 1703091554&sprefix = L298N+%2Caps%2C262&sr = 8–4
Old magnetic hard disk drive		1	$0	$0	

## 5 Build instructions

**Fig. 4:**
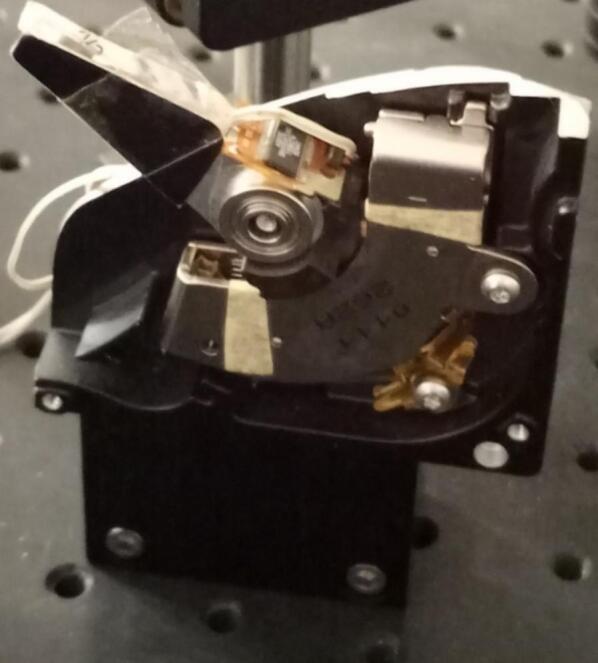
The mechanical shutter made by a voice coil hard disk drive mechanism.

To reproduce this setup with the shutter, one should acquire an old hard disk, that can be easily taken from an old desktop computer. The building indications is more detailed for the building of the controller, since the mechanical shutter is only composed of the driver module and the voice-coil actuator (Fig. 4).

### 5.1 Build the mechanical shutter

• The first thing to do is cutting the housing of the hard disk while preserving the coil-motor mechanism
• Identify the two ends of the coil, which are just on the side of the pivot arm.
• Connect the two ends of the coil to the outputs Out1 and Out2 of the H Bridge driver, L298N module

### 5.2 Build the controller

The main purpose of the controller is to generate the different pulses that controls the triggering of the command signals with the shutter opening/closing time. The main parameter here is knowing the values of the period T of the trigger signal which will be used; this will govern the choice of the capacitors (C_1_ and C_7_) values and potentiometers values. The values in the bill material are valid for the used case of T=10Hz as explained in the design section.

**Fig. 5:**
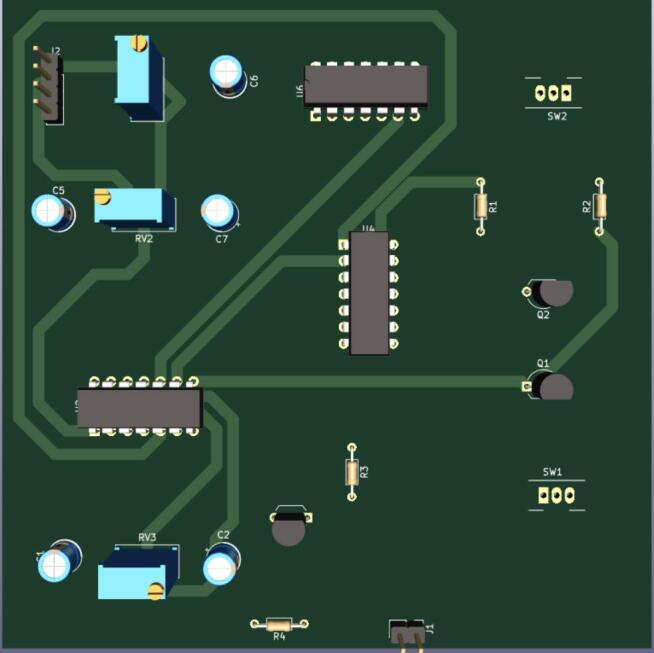
FriiTriga pcb 3D view.

• Collect the items listed on the bill of materials, and tune the potentiometers RV2 and RV3 according to the period T of the trigger signal with respects to the following equations:
  o 1RV3×C1=0.5×T
  o 1RV2×C7=n+0.5×T with n being the number of pulses one wants the shutter to let out. n defines, in other words, the opening time $\tau_{open}$ of the shutter which is $\tau_{open}=n\times T$.
• Realize the circuit board according to schematic in the design file FriiTriga_KiCAD_Design.zip. One can use the editable KiCAD schematic file and produce a KiCAD layout for a professional PCB (Fig. 5).

**Fig. 6:**
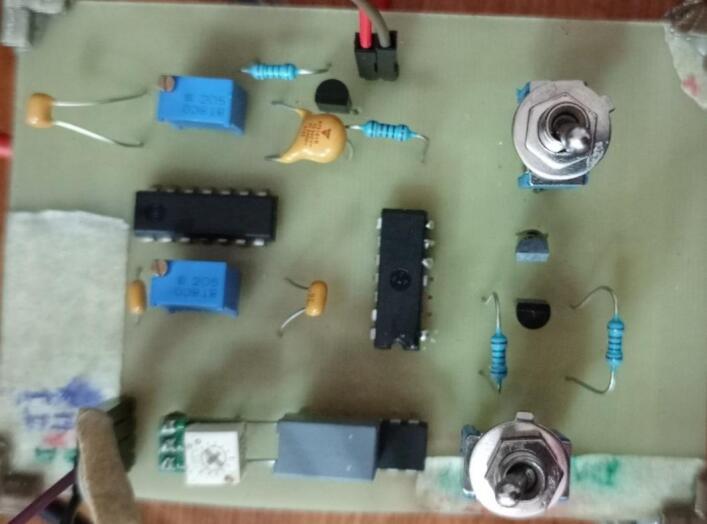
Manually made friitriga controller pcb card.
• Now the last step is connecting the different input signals of the circuit board following the numbering written on the schematic file:
  o Connect the trigger signal ground, the logic signal command ground and the L298N module ground to Pin1 of J2
  o Connect Pin2 of J2 to the 5 V output of the L298N module
  o Connect Pin3 to the logic signal command (it’s this signal which will order the opening or closing of the shutter)
  o Connect Pin4 to the trigger signal (laser trigger or any periodic signal one wants to use as trigger)
• After these steps, the circuit is ready to be used. There are two switches that define manual operation, direct command operation or triggered operation:
  o For manual operation: one just needs to use the switch SW1; one position will close the shutter and the other will open it.
  o For direct command operation: SW1 has to be on the position where the shutter is closed, then the switch SW2 will be placed on position mentioned Pos_D (as mentioned on the schematic file). Now the shutter will open when a command signal is sent and the opening time will be the signal high state time duration.
  o For triggered operation, the switch SW1 has to be kept the close position, and SW2 pushed on Pos_T (as mentioned on the schematic file). Then one sends a signal command which high state duration is equal to the trigger period T. The shutter will then be opened according to the tuning which have been made on RV2 previously.
• One last thing to notice is that, if one wants to change the opening time of the shutter during the triggered operation, this can be done at any moment by just tuning RV2 and visualize on an oscilloscope the pin 1 of the 74HC86 IC to respect the timing equations.

In this study, the pcb has been made manually, as showed in the following image:

• For those who do not know how to use the motor driver L298N, you can visit this instructable https://www.instructables.com/Arduino-Modules-L298N-Dual-H-Bridge-Motor-Controll/
• Connect the outputs Output1 and Output2 (connector J1 on the schematic file) of the circuit board to the inputs IN1 and IN2 of the L298N module.
• At the power input VIN of the L298N module connect a power source which value is between 6 V and 8 V (it’s the tension needed to switch the shutter, yours may be slightly different). In this study case, it has been noticed that a power source higher than 8 V causes excess heat in the voice coil actuator. Do not forget to connect the negative pin of the power source to the input GND of the L298N module (Fig. 6).

## 6 Operation instructions

**Fig. 7:**
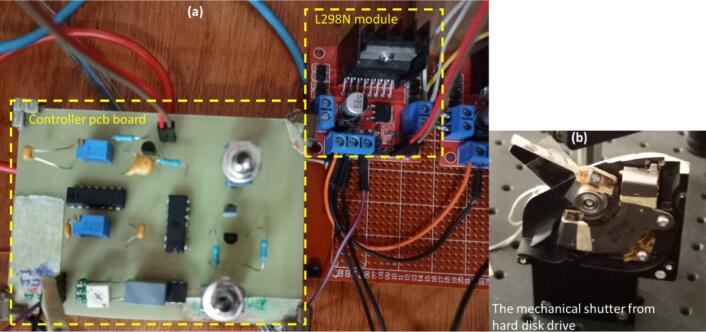
The complete setup showing (a) the friitriga controller and the l298n module, and (b) the mechanical shutter made from hard disk drive.

The use of the setup is very simple. The first thing is to define how the command signal will be sent. In this study case, a microcontroller ESP32 was used to generate the TTL command signal. Fig. 7 present the complete setup:

• A high state logical signal pulse is sent to the Command input of the controller board, the duration of the pulse is equal of the opening time of the shutter when in direct mode; or the pulse duration is equal to the trigger period and the opening time depends on how you tune RV2 potentiometer.
• The actuator will move for the required duration time.
• The mechanical switches SW1 and SW2 are there for the different user mode, as described in the previous section.

## 7 Validation and characterization

The triggered shutter was characterized using a silicon (Si) based photodetector. Four parameters were tackled on this measurement: the opening time, the closing time, the minimum open pulse, and the delay response time.

For the measurements, the Si detector is aligned to a broadband light source and connected to an oscilloscope; the shutter is placed between the detector and the light source and blocked the light path. The oscilloscope is triggered for single measurement so that the rising of the signal when the shutter will be opened can be captured. The same process was repeated to capture the closing time of the shutter.

**Fig. 8:**
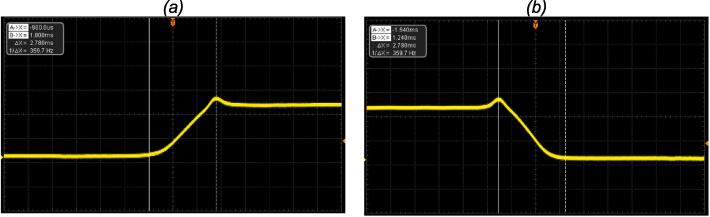
Manual mode characterization, showing the Si detector signal (down is shutter closed, and up is shutter opened): (a) measurement of the opening time and (b) measurement of the closing time of the shutter.

For the measurement of the minimum open pulse, different command signals pulses durations were provided in direct mode (starting from 1 ms) to the setup, and the signal was measured at the oscilloscope for a shutter opening for one (01) pulse time (Fig. 8).

**Fig. 9:**
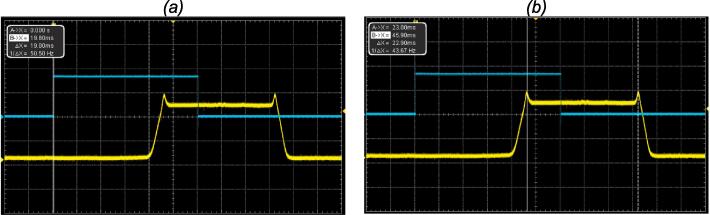
Direct mode measurements showing the command signal in blue and the Si detector signal in yellow. (a) measurement of the delay between the command signal and the shutter opening in direct mode; (b) measurement of the minimal opening time. (For interpretation of the references to colour in this figure legend, the reader is referred to the web version of this article.)

**Fig. 10:**
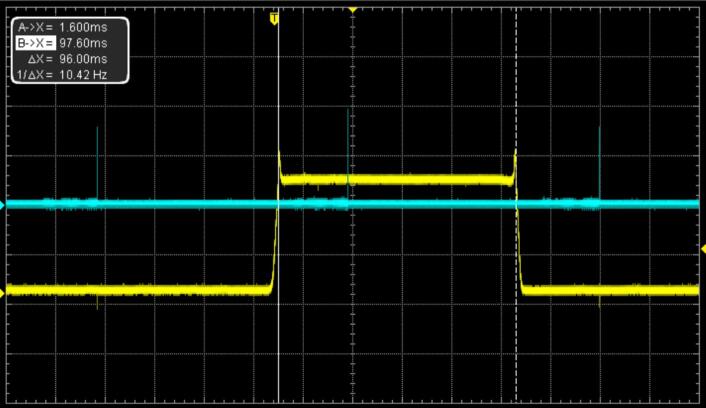
Triggered mode characterization showing the trigger signal in blue, which has a frequency of 10 Hz, and the detector signal indicating the opening of the shutter. (For interpretation of the references to colour in this figure legend, the reader is referred to the web version of this article.)

The opening and closing time measured was the same for all the different user modes. Table 1 present a resume of the characteristics measurements compared with the equivalent commercial component from Thorlabs (SH05 shutter with KSC101 controller). The setup presented in this study offer better characteristics for the opening and closing times; the minimum open times are almost the same, and the Thorlabs component offer better characteristic on the delay response time (Fig. 9 and Fig. 10).

**Table 1:** FriiTriga timing characteristics compared with the Thorlabs lowest cost shutter (SH05) and controller (KSC101).

Parameter measured	Value of this study	Thorlabs shutter SH05 with KSC101 controller [1]
Opening Time	3 ms	5.5 ms
Closing Time	3 ms	8 ms
Delay Response Time	19 ms	11 ms
Minimum Opening Time	23 ms	24 ms

**Fig. 11:**
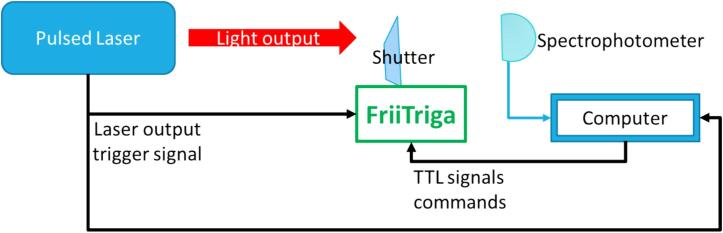
Example of application of FriiTriga controller for synchronization of spectrophotometer measurements with laser output.

As an example of application, the FriiTriga controller presented in this study, was developed and used in an optical setup for laser induced spectroscopy. The FriiTriga is used to synchronize the opening of the shutter with the pulsed laser trigger signal. The controller was tuned as described in the case study of Section 2. Thanks to the FriiTriga, the spectrophotometer measurement is synchronized with the arrivals of laser pulses (controlled by the opening/closing of the shutter). The synoptic description of this use case is described in Fig. 11.

## CRediT authorship contribution statement

**Dam-Bé L. Douti:** . **Chérif Ouro-Wassara:** Visualization, Software, Data curation. **Essohana Mabafei:** Visualization, Software, Data curation.

## Declaration of competing interest

The authors declare that they have no known competing financial interests or personal relationships that could have appeared to influence the work reported in this paper.
